# Efficacy and Safety Associated With Immune Checkpoint Inhibitors in Unresectable Hepatocellular Carcinoma: A Meta-analysis

**DOI:** 10.1001/jamanetworkopen.2021.36128

**Published:** 2021-12-06

**Authors:** Alexandre A. Jácome, Ana Carolina G. Castro, João Paulo S. Vasconcelos, Maria Helena C. R. Silva, Marco Antônio O. Lessa, Eduardo D. Moraes, Aline C. Andrade, Frederico M. T. Lima, João Paulo F. Farias, Roberto A. Gil, Gabriel Prolla, Bernardo Garicochea

**Affiliations:** 1 Department of Gastrointestinal Medical Oncology, Oncoclínicas, Belo Horizonte, Brazil; 2 Oncoclínicas, Salvador, Brazil; 3 Oncoclínicas, Rio de Janeiro, Brazil; 4 Oncoclínicas, Porto Alegre, Brazil; 5 Oncoclínicas, São Paulo, Brazil

## Abstract

**Importance:**

Immune checkpoint inhibitors (ICIs) have yielded conflicting results in hepatocellular carcinoma (HCC). The overall effect of ICIs compared with standard therapies in unresectable HCC requires more research.

**Objective:**

To estimate the efficacy and safety associated with ICIs compared with standard therapies in patients with unresectable HCC.

**Data Sources:**

PubMed, Cochrane Library, Web of Science, Latin American and Caribbean Health Sciences Literature, and American Society of Clinical Oncology and European Society of Medical Oncology meeting proceedings were systematically searched. Reference lists from studies selected by electronic searching were manually searched to identify additional relevant studies. The search included literature published or presented from February 2010 to February 2020.

**Study Selection:**

From December 2019 to February 2020, independent reviewers evaluated each database, scanning the title, abstract, and keywords of every record retrieved. Full articles were further assessed if the information given suggested that the study was a randomized clinical trial (RCT) comparing ICIs vs standard therapies in the treatment of unresectable HCC.

**Data Extraction and Synthesis:**

The full text of the resulting studies and extracted data were reviewed independently according to PRISMA guidelines. Summary hazard ratios (HRs) of overall survival (OS) and progression-free survival (PFS) were calculated by a random-effects model. The likelihood of ICIs being associated with overall response rate (ORR) and treatment-related adverse events (TRAEs) was expressed by odds ratios (ORs) using a random-effects model.

**Main Outcomes and Measures:**

The main outcomes were OS, PFS, ORR, and TRAEs.

**Results:**

Of 1836 studies yielded by the search, 3 were retained, totaling 1657 patients (985 treated with ICIs vs 672 receiving standard treatment). Two studies evaluated ICIs as monotherapy, and 1 study investigated the combination of ICIs with bevacizumab. Compared with standard therapies (sorafenib in first-line therapy or placebo in second-line therapy), ICIs were associated with significantly improved OS (HR, 0.75; 95% CI, 0.62-0.92; *P* = .006), PFS (HR, 0.74; 95% CI, 0.56-0.97; *P* = .03), and ORR (OR, 2.82; 95% CI 2.02-3.93; *P* < .001). The probability of grade 3 or 4 TRAEs was lower with ICIs than with sorafenib (OR, 0.44; 95% CI, 0.20-0.96; *P* = .04).

**Conclusions and Relevance:**

This meta-analysis found superior efficacy and safety associated with ICIs compared with standard therapies and highlights the survival benefit associated with the combination of antiangiogenic therapy with ICIs in first-line systemic therapy of unresectable HCC.

## Key Points

**Question:**

What are the efficacy and safety associated with immune checkpoint inhibitors (ICIs) vs standard therapies in unresectable hepatocellular carcinoma (HCC)?

**Findings:**

In a meta-analysis of 3 randomized clinical trials totaling 1657 patients, ICIs were associated with significantly improved overall survival, progression-free survival, and overall response rate compared with standard therapies. In addition, the rate of grade 3 or 4 treatment-related adverse events was lower with ICIs than with sorafenib.

**Meaning:**

These findings suggest that ICIs should be the new standard of care in systemic therapy of unresectable HCC.

## Teaser

This meta-analysis assesses the overall treatment outcome associated with ICIs compared with standard therapies in patients with unresectable hepatocellular carcinoma.

## Introduction

Hepatocellular carcinoma (HCC) is one of the most lethal malignant neoplasms, ranking as the fourth most common cause of cancer-related death in the world.^1^ Approximately 20% of patients have advanced disease at presentation, which portends a poor prognosis, with an estimated 5-year overall survival (OS) rate of 2%.^2^

The cornerstone of the treatment of unresectable or metastatic HCC is systemic therapy. Since 2008, sorafenib has been the standard of care, based on the pivotal phase 3 SHARP trial, which demonstrated an improvement in the median OS vs placebo (10.7 months vs 7.9 months; hazard ratio [HR], 0.69, 95% CI, 0.55 to 0.87; *P* < .001).^3^ In the past few years, other antiangiogenic agents, either tyrosine-kinase inhibitors or monoclonal antibodies, have been found to be effective for both patients who are treatment-naive (lenvatinib,^4^ donafenib^5^) and those who are resistant to sorafenib-based treatment (cabozantinib,^6^ regorafenib,^7^ apatinib,^8^ and ramucirumab^9^). Nevertheless, none has shown superiority over sorafenib.

Immune checkpoint inhibitors (ICIs) have ushered in a new era in cancer therapy, but their efficacy in HCC is uncertain. Single-arm phase 2 studies with patients who are resistant to sorafenib suggested clinical activity of nivolumab,^10^ pembrolizumab,^11^ and the combination of ipilimumab plus nivolumab,^12^ all of which have been approved by the US Food and Drug Administration. When evaluated as monotherapy in randomized clinical trials (RCTs), anti–programmed cell death protein 1 (PD-1) and anti–programmed cell death 1 ligand 1 (PD-L1) inhibitors did not demonstrate superiority compared with standard care. Nivolumab and sorafenib yielded similar OS rates in previously untreated patients (16.4 months vs 14.7 months; HR, 0.85; 95% CI, 0.72 to 1.02; *P* = .08),^13^ and pembrolizumab did not meet the co–primary end point of OS in a placebo-controlled trial (13.9 months vs 10.6 months for placebo; HR, 0.78; 95% CI, 0.61 to 0.99; *P* = .02).^14^

The combination of atezolizumab, an anti–PD-L1 inhibitor, with bevacizumab was compared with sorafenib as first-line treatment of unresectable HCC in the phase 3 IMbrave150 trial.^15^ The combination was superior to sorafenib in OS and progression-free survival (PFS), the co–primary end points (12-month OS, 67.2% vs 54.6%; HR, 0.58; 95% CI, 0.42-0.79; *P* < .001; median PFS, 6.8 months vs 4.3 months; HR, 0.59; 95% CI, 0.47-0.76; *P* < .001).^15,16^ Based on the IMbrave150 findings, the combination of atezolizumab plus bevacizumab has replaced sorafenib as the standard of care for unresectable HCC.

Owing to the conflicting results of the studies with immunotherapy in HCC, we performed, to our knowledge, the first meta-analysis of RCTs addressing the overall outcomes associated with ICIs compared with the standard of care in unresectable HCC.

## Methods

This meta-analysis did nor require institutional review board approval because the sources of the analyzed data are public and the analysis will not make the data individually identifiable. This review was conducted according to the Preferred Reporting Items for Systematic Reviews and Meta-analyses (PRISMA) reporting guideline and is registered in PROSPERO (CRD42020162599).

### Search Strategy

We systematically searched for studies in PubMed/MEDLINE, the Cochrane Central Register of Controlled Trials, Web of Science, Latin American and Caribbean Health Sciences Literature (LILACS), and European Society of Medical Oncology (ESMO) and American Society of Clinical Oncology (ASCO) proceedings using the following search strategy: (*liver* OR *hepatic* OR hepato* [all fields]) AND (*cancer* OR *carcinoma* OR *adenocarcinoma* OR *tumor* OR *tumour* OR *malignant* OR *neoplasm* [all fields]) AND (*checkpoint* OR *nivolumab* OR *pembrolizumab* OR *cemiplimab* OR *atezolizumab* OR *avelumab* OR *durvalumab* OR *ipilimumab* OR *tremelimumab* OR *anti-PD-1* OR *anti-PD-L1* OR *anti-CTLA-4* OR immun* [all fields]) AND (*survival* [all fields]).

Reference lists from studies selected by electronic searching were manually searched to identify additional relevant studies. The search was performed between December 2019 and February 2020, and included literature published or presented up to February 2020.

### Selection Criteria

Articles were included if they were RCTs that compared the OS of patients with unresectable HCC treated with ICIs vs standard care, regardless of the therapeutic line. We searched for studies published in the past 10 years, with no language restriction.

To select studies for further assessment, 2 or 3 independent reviewers evaluated each of the 6 databases, scanning the title, abstract, and keywords of every record retrieved. Full articles were further assessed if the information given suggested that the study was an RCT comparing ICIs vs standard care in the treatment of advanced HCC.

### Data Extraction

Two of us (A.A.J. and A.C.G.C.) reviewed the full text of the 3 resulting studies and extracted data independently. Data were extracted at the trial level and not the individual patient data level owing to lack of availability. Any differences in extracted data were resolved via consensus between us. If a consensus could not be reached within the pair, the entire author group was consulted to achieve consensus on the most accurate results. Extracted data included details on study design, inclusion and exclusion criteria, randomization, interventions, experimental and comparative drugs, the therapeutic line, number of patients in each arm, outcomes measured (OS, PFS, overall response rate [ORR], grade 3-4 treatment-related adverse events [TRAEs]), and results (number of events, HRs, ORs, 95% CIs, and *P* values). The HRs of time-to-event variables (OS and PFS) were directly extracted from the original studies or were estimated indirectly using the reported number of events and the corresponding *P* value for the log-rank statistics. Data were extracted using an assessment form that was designed specifically for the topic of this study.

### Statistical Analysis

Outcomes of interest included OS, PFS, ORR, and grade 3-4 TRAEs. A summary HR of OS and PFS was calculated using a 95% CI by a random-effects model. The likelihood of ICIs being associated with ORR and TRAEs was expressed by OR and 95% CI using a random-effects model and presented in forest plots. The treatment outcome for each study was expressed as a ratio of the ICIs group vs the standard care group. The quality of the trials was evaluated by the Cochrane risk-of-bias tool for randomized trials.^17^ Statistical heterogeneity in the results of the trials was assessed by the χ^2^ test and was expressed by the *I*^2^ index, as described by Higgins and colleagues.^18^ Publication bias was evaluated with Egger test.^19^

Statistical analysis of summary data was performed with RevMan software version 5.3 (Cochrane). Hypotheses tests were 2-sided and *P* < .05 was deemed as statistically significant.

## Results

**Figure 1.**
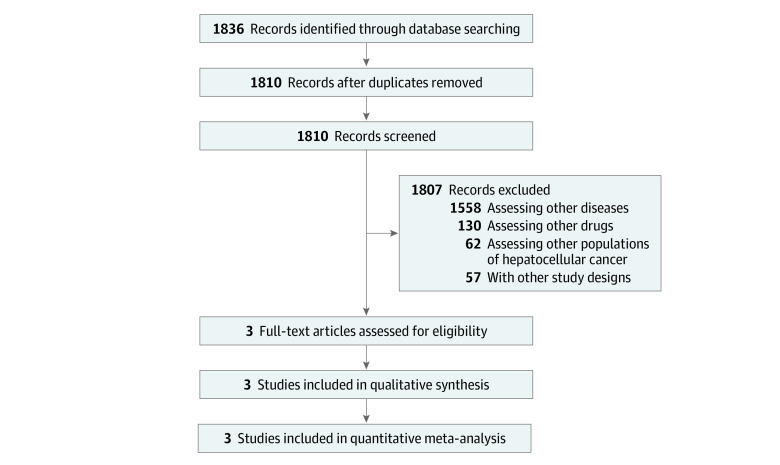
Study Selection Flowchart

Of the 1836 studies yielded by the search, 3 studies were retained, totaling 1657 patients (985 ICIs vs 672 standard care): KEYNOTE-240,^14^ CheckMate-459,^13^ and IMbrave150^15,16^ (Table and Figure 1). Two studies compared ICIs vs sorafenib in the first-line setting (CheckMate-459 and IMbrave150), and 1 study compared an ICI with placebo in the second-line setting (KEYNOTE-240). All 3 studies were phase 3 RCTs. The ICIs studied were nivolumab in KEYNOTE-240, atezolizumab in CheckMate-459, and pembrolizumab in IMbrave150. RCTs with anti-CTLA4 have not been reported, to our knowledge. Two studies evaluated ICIs as monotherapy (CheckMate-459 and KEYNOTE-240), and 1 study investigated an ICI in combination with bevacizumab (IMbrave150).

**Table.** Summary of the Studies Selected

Source	No.		ORR, %		PFS				OS				Grade 3-4 TRAE, %		Systemic therapy		Patients with HBV or HCV, %	Primary end point	Crossover permitted
	ICI	SC	ICI	SC	Median, mo		HR (95% CI)	*P* value	Median, mo		HR (95% CI)	*P* value	ICI	SC	ICI	SC			
					ICI	SC			ICI	SC									
Finn et al,^14^ 2019	278	135	18	4	3.0	2.8	0.71 (0.57-0.90)	.002	13.9	10.6	0.78 (0.61-0.99)	.02	19	8	Pembrolizumab	Placebo	40	OS, PFS	Yes
Yau et al,^13^ 2019	371	372	15	7	3.7	3.8	0.93 (0.79-1.10)	NR	16.4	14.7	0.85 (0.72-1.02)	.07	22	49	Nivolumab	Sorafenib	54	OS	Yes
Cheng et al,^15,16^ 2019	336	165	27	12	6.8	4.3	0.59 (0.47-0.76)	<.001	NR	13.2	0.58 (0.42-0.79)	<.001	36	46	Atezolizumab + bevacizumab	Sorafenib	69	OS, PFS	Yes

Abbreviations: HBV, Hepatitis B virus; HCV, Hepatitis C virus; HR, hazard ratio; ICI, immune checkpoint inhibitor; NR, not reported; ORR, overall response rate; OS, overall survival; PFS, progression-free survival; SC, standard care; TRAE, treatment-related adverse event.

### OS

In KEYNOTE-240,^14^ the median OS was 13.9 months in the pembrolizumab group vs 10.6 months in the placebo group (HR, 0.78; 95% CI, 0.61-0.99; *P* = .02) (Table). However, OS did not meet KEYNOTE-240’s prespecified boundary of *P* = .0174 for significance in the final analysis. At disease progression in each group, systemic anticancer therapies were used by 41.7% of patients in the ICI group vs 47.4% of patients in the placebo group. Crossover was allowed.

Likewise, in CheckMate-459, OS did not meet the predefined threshold of statistical significance (HR, 0.84; *P* = .04).^13^ The median OS was 16.4 months in the nivolumab group vs 14.7 months in the sorafenib group (HR, 0.85; 95% CI, 0.72-1.02; *P* = .07). The 24-month OS rate was 36.8% in the nivolumab group vs 33.1% in the sorafenib group. At disease progression, patients could receive subsequent therapies at the investigator’s discretion. Among patients initially enrolled in the sorafenib group, 26% subsequently received immunotherapy.

IMbrave150 reached the primary end point: the 12-month OS rate was 67.2% in the atezolizumab plus bevacizumab group and 54.6% in the sorafenib group (HR, 0.58; 95% CI, 0.42-0.79; *P* < .001).^15,16^ As of the time of the study’s primary analysis (August 29, 2019), the median OS in the ICI group had not been reached. The sorafenib group had a median OS of 13.2 months. Crossover was also allowed.

**Figure 2.**
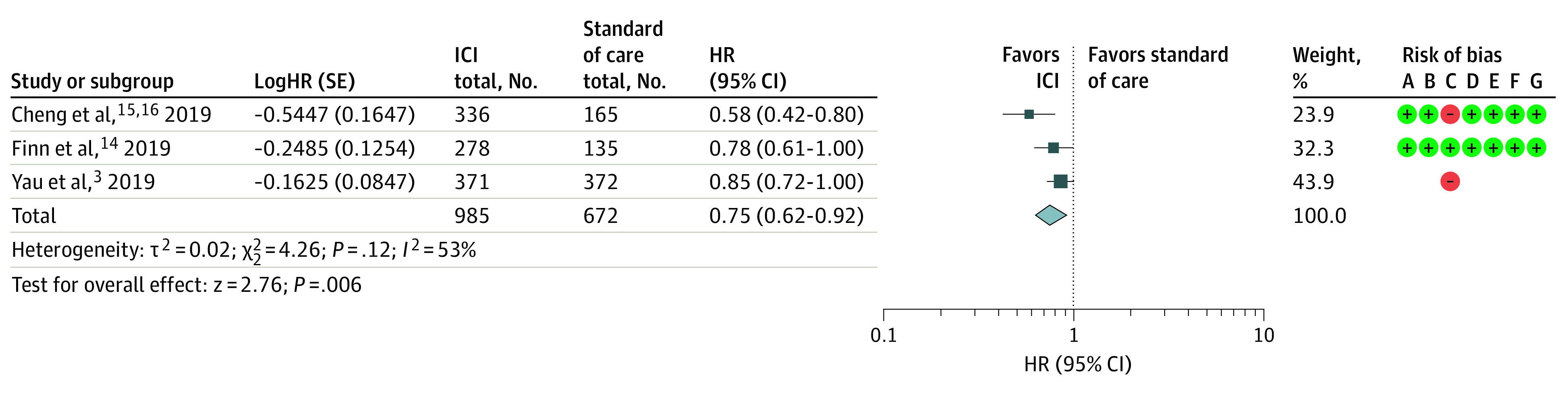
Assessment of Overall Survival The diamond indicates best estimate of the true (pooled) outcome (with width indicating 95% CI); HR, hazard ratio; ICI, immune checkpoint inhibitor. Risks of bias are present (+) or absent (-), with A indicating random sequence generation (selection bias); B, allocation concealment (selection bias); C, blinding of participants and personnel (performance bias); D, blinding of outcome assessment (selection bias); E, incomplete outcome data (attrition bias); F, Selective reporting (reporting bias); and G, other bias.

Compared with standard care (sorafenib in first-line or placebo in second-line), ICIs were associated with significantly improved OS (HR, 0.75; 95% CI, 0.62-0.92; *P* = .006) (Figure 2). No significant heterogeneity was found among the studies (*I*^2^ = 53%), and the funnel plot showed no evidence of publication bias (eFigure 1 in the Supplement).

### PFS

Compared with the placebo, pembrolizumab increased the median PFS in KEYNOTE-240 at the final analysis: 3.0 months vs 2.8 months (HR, 0.71; 95% CI, 0.57-0.90, *P* = .0022).^14^ At the first interim analysis, the median PFS was similar: 3.0 months vs 2.8 months (HR, 0.77; 95% CI, 0.60-0.98; *P* = .01). Similar to OS, the PFS at the first interim analysis, the coprimary end point, did not meet the prespecified boundary (*P* = .002). Disease progression was evaluated via blinded and independent central radiologic review according to Response Evaluation Criteria in Solid Tumors (RECIST) version 1.1.

PFS was not a primary end point in CheckMate-459. The median PFS was similar between the nivolumab and sorafenib groups: 3.7 months vs 3.8 months (HR, 0.93; 95% CI, 0.79-1.10).^13^ The 12-month PFS rate was 22% in the nivolumab group vs 14% in the sorafenib group.

The highest PFS was observed in IMbrave150: 6.8 months in the atezolizumab plus bevacizumab arm, compared with 4.3 months in the sorafenib group (HR, 0.59; 95% CI, 0.47-0.76; *P* < .001).^15,16^ Disease progression was based on RECIST, as assessed at an independent review facility.

**Figure 3.**
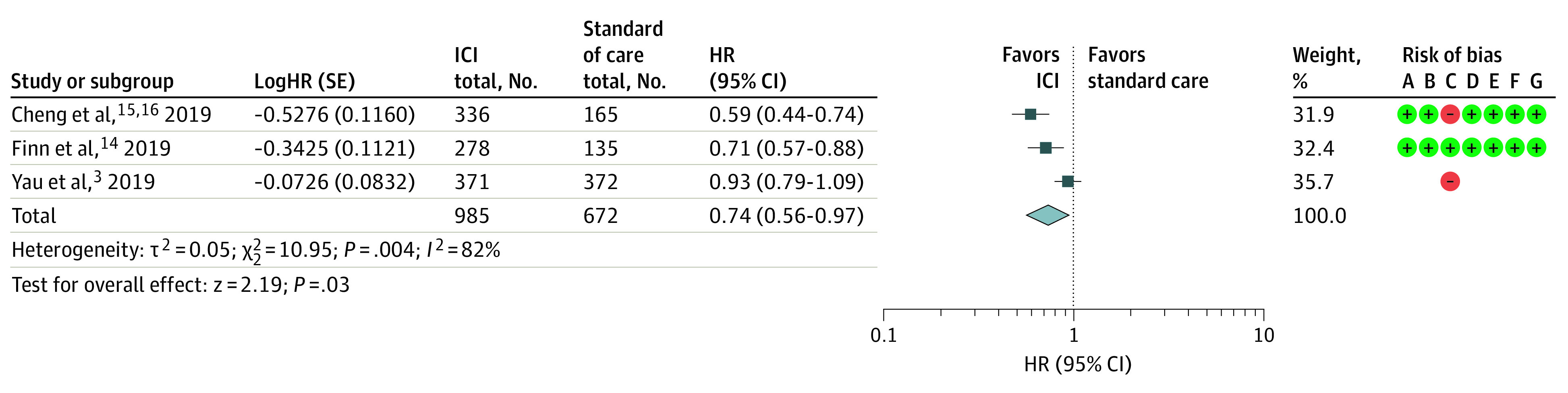
Assessment of Progression-Free Survival The diamond indicates best estimate of the true (pooled) outcome (with width indicating 95% CI); HR, hazard ratio; ICI, immune checkpoint inhibitor. Risks of bias are present (+) or absent (-), with A indicating random sequence generation (selection bias); B, allocation concealment (selection bias); C, blinding of participants and personnel (performance bias); D, blinding of outcome assessment (selection bias); E, incomplete outcome data (attrition bias); F, Selective reporting (reporting bias); and G, other bias.

Treatment with ICIs was associated with greater improvement in PFS compared with standard care (HR, 0.74; 95% CI, 0.56-0.97; *P* = .03) (Figure 3). Significant heterogeneity was found among the studies in the analysis of PFS (*I*^2^ = 82%).

### ORR

All patients included in all 3 studies had measurable disease, and response evaluation was performed by an independent central review per RECIST 1.1 in all studies. In KEYNOTE-240, the ORR was 4% in the placebo group and 18% in the pembrolizumab arm group (*P* < .001).^14^ Six patients (2.2%) who received the ICI reached complete response.

Likewise, in CheckMate-459, the ORR was higher in the nivolumab group (15%) than in the sorafenib group (7%) (HR, 2.41; 95% CI, 1.48-3.92).^13^ Patients with PD-L1 expression at 1% or greater had a higher ORR than their patients without PD-L1 expression: 28% among the nivolumab group vs 9% among the sorafenib group of patients with PD-L1 expression, compared with 12% among the nivolumab group vs 7% the sorafenib group among patients without PD-L1 expression.

The ORR was also higher in patients treated with atezolizumab plus bevacizumab than in those treated with sorafenib: 27% vs 12% (*P* < .001).^15,16^ The difference in ORR between groups was higher when response was evaluated per HCC-specific modified RECIST: 33% vs 13% (*P* < .001). Based on RECIST, 5.5% of the patients who received combination therapy reached complete response, compared to 10.2% based on HCC-specific modified RECIST.

**Figure 4.**
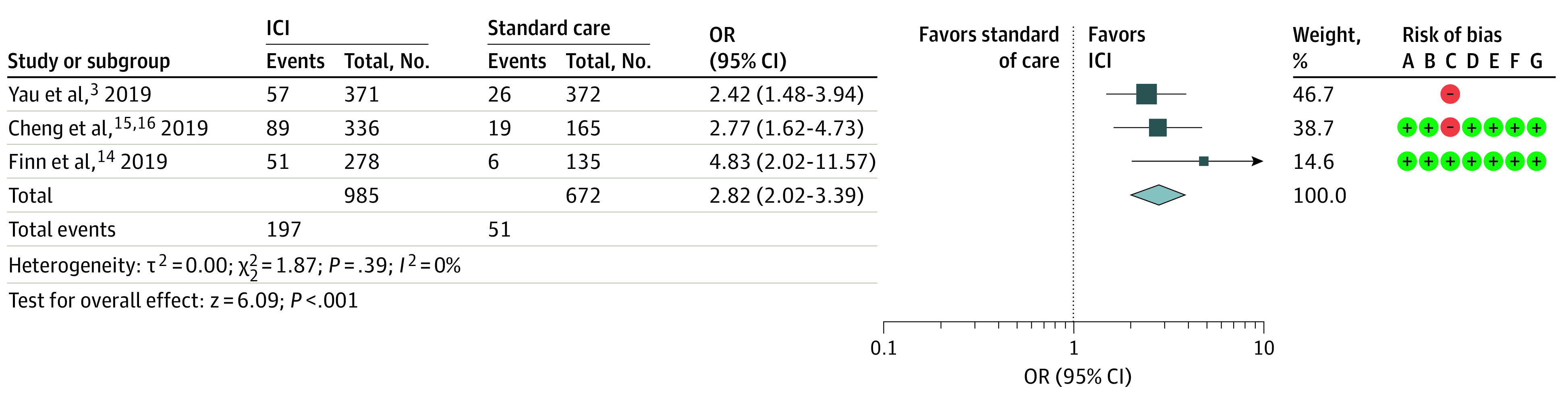
Assessment of Overall Response Rate The diamond indicates best estimate of the true (pooled) outcome (with width indicating 95% CI); ICI, immune checkpoint inhibitor; OR, odds ratio. Risks of bias are present (+) or absent (-), with A indicating random sequence generation (selection bias); B, allocation concealment (selection bias); C, blinding of participants and personnel (performance bias); D, blinding of outcome assessment (selection bias); E, incomplete outcome data (attrition bias); F, Selective reporting (reporting bias); and G, other bias.

Based on RECIST, treatment with ICIs was associated with better ORR than standard care (sorafenib in first-line or placebo in second-line) (OR, 2.82; 95% CI, 2.02-3.93; *P* < .001) (Figure 4). No significant heterogeneity was found among the studies (*I*^2^ = 0%).

### TRAEs

The rate of grade 3 or 4 TRAEs was lower in patients who received ICIs than in those who received sorafenib. In CheckMate-459, the rate of grade 3 or 4 TRAEs was 22% in patients who received nivolumab monotherapy and 49% in those treated with sorafenib.^13^ Cutaneous reactions were the main TRAE in patients who received sorafenib (2% of patients who received nivolumab vs 18% of patients who received sorafenib), while hepatic AEs represented the major issue for those who received nivolumab (10% of patients who received nivolumab vs 7% of patients who received sorafenib). Grade 3 or 4 TRAEs led to treatment discontinuation in 4% of patients who received nivolumab and 8% of patients who received sorafenib of patients.

Likewise, in IMbrave150, the rate of grade 3 or 4 TRAEs was 46% in the sorafenib group and 36% in the atezolizumab plus bevacizumab group.^15,16^ The percentage of patients who discontinued any treatment component because of AEs was 16% in the combination group (7% discontinued both components) and 10% in the sorafenib group. The most frequent grade 3 or 4 TRAE in both groups was hypertension, present in 15% in the ICI group vs 12% in the sorafenib group. The next most frequent was aspartate aminotransferase increase, present in 7% of patients who received ICIs vs 5% of patients who received sorafenib.

In KEYNOTE-240, the rate of grade 3 or 4 TRAEs was 8% in the placebo group and 19% in the pembrolizumab group.^14^ Aspartate aminotransferase increase(13% of patients) and hyperbilirubinemia (8% of patients) were the most frequent grade 3 or 4 AEs in the pembrolizumab group. KEYNOTE-240 data were not included in the overall analysis of TRAEs owing to the comparison with placebo.

Overall, the probability of grade 3 or 4 TRAEs was lower with ICIs than with sorafenib (OR, 0.44; 95% CI, 0.20-0.96; *P* = .04) (eFigure 2 in the Supplement). Significant heterogeneity was found among the studies (*I*^2^ = 89%).

## Discussion

In this meta-analysis of 3 RCTs evaluating 1657 patients with unresectable HCC, in both the first-line and second-line setting, ICIs were shown to be associated with significantly better ORR, PFS, and OS, which had an overall reduction of 25% in the relative risk of death, compared with standard care (sorafenib or placebo). In addition, ICIs had a safer toxicity profile, with lower rates of grade 3 or 4 TRAEs.

The main driver of the overall benefit associated with ICIs found in this meta-analysis was the data obtained from the IMbrave150 study, which evaluated the combination of anti–PD-L1 plus anti–vascular endothelial growth factor (VEGF) therapy.^15,16^ ICIs as monotherapy have demonstrated limited efficacy in the systemic treatment of HCC. Since OS may be influenced by subsequent therapies and crossover, PFS might be a more unbiased end point to analyze the efficacy of ICIs. In CheckMate-459, PFS was similar between nivolumab and sorafenib (3.7 months vs 3.8 months),^13^ and in KEYNOTE-240, pembrolizumab yielded an improvement in PFS compared with placebo (3.0 months vs 2.8 months; HR, 0.71; 95% CI, 0.57-0.90; *P* = .0022).^14^ The phase 1b trial GO30140 by Lee et al^21^ randomly assigned previously untreated patients with unresectable HCC to atezolizumab plus bevacizumab or atezolizumab alone. In concordance with CheckMate-459^13^ and KEYNOTE-240,^14^ the GO30140 trial demonstrated shorter PFS with atezolizumab monotherapy than with the dual therapy (3.4 months vs 5.6 months; HR, 0.55; 80% CI 0.40-0.74; *P* = .01), with a median follow-up of 6.6 months. On the other hand, both IMbrave150 (HR, 0.59)^15^ and GO30140 (HR, 0.55)^21^ confirmed a clear superiority in PFS of the combination of anti–PD-L1 plus anti-VEGF compared with either anti–PD-L1 monotherapy or anti-VEGF alone.

Our data corroborate the findings of a recently published network meta-analysis that compared the efficacy of ICIs, VEGF inhibitors, and their combination among phase 3 clinical trials in patients with advanced HCC.^22^ It found that dual therapy with atezolizumab plus bevacizumab was associated with better outcomes than monotherapy with either nivolumab (HR, 0.68; 95% CI, 0.48-0.98) or VEGF inhibitors, such as lenvatinib (HR, 0.63; 95% CI, 0.44-0.89) and sorafenib (HR, 0.58; 95% CI, 0.42-0.80).^22^ That meta-analysis and ours support the conclusion that PFS and OS are similar between ICIs and VEGF inhibitors as first-line monotherapy. In a further analysis, our study found that ICIs were associated with improved ORR (OR, 2.82) and reduced toxic effects, corresponding to a reduction of 56% in the risk of grade 3 or 4 TRAEs compared with sorafenib.

Nevertheless, most patients with HCC do not have an objective response to ICIs, even in combination therapies.^15,23^ HCC is a markedly heterogenous disease, frequently representing end-stage liver disease secondary to quite distinct underlying causes. It is not clear which patients are more sensitive to immunotherapeutic approaches. CheckMate-459 presented efficacy data by PD-L1 expression and suggested that patients with expression at 1% or greater had a higher likelihood to present ORR to nivolumab: 28% vs 9% with sorafenib in patients with PD-L1 expression, compared with 12% vs 7% in patients without PD-L1 expression.^13^ Patients with HCC associated with viral hepatitis also were more sensitive to ICIs. Chronic inflammation induces the expression of immune checkpoint molecules and promotes effector T-cell exhaustion,^24^ which might explain the supposed higher sensitivity of patients infected with hepatitis B or hepatitis C virus. Among 3 randomized clinical trials included in our study, IMbrave150 had the highest proportion of patients with viral hepatitis, representing 69% of the overall population,^15^ compared with 54% in CheckMate-459^13^ and 40% in KEYNOTE-240.^14^ However, correlating the higher degree of benefit of the dual therapy to the higher proportion of patients with viral hepatitis would be speculative.

### Limitations

Our meta-analysis has some limitations. The strict inclusion criteria of our systematic review allowed for the selection of only 3 studies. Despite being an RCT addressing the role of ICIs, the GO30140 trial^21^ did not meet the criteria for being included in our meta-analysis because the comparator group (atezolizumab as monotherapy in the first-line setting) is not standard care. There is no minimum number of studies to be analyzed in a meta-analysis. However, meta-analyses with few studies or with small sample sizes compromise the precision of the tests that measure heterogeneity, such as τ^2^, χ^2^, and *I*^2^.^25^ On the other hand, since ours is the first meta-analysis, to our knowledge, to analyze the efficacy and safety of ICIs compared with standard care in unresectable HCC, which is an innovative field, it is expected to find a low number of RCTs.

The diversity of the RCTs analyzed in our meta-analysis prompted us to choose the random-effects model, since we do not expect a fixed effect in clinical trials with different control (sorafenib or placebo) and experimental (ICIs alone or ICI plus bevacizumab) groups. The inclusion of the IMbrave150 was the main source of between-study heterogeneity, as can be seen through the analysis of the funnel plot (eFigure 1 in the Supplement), which helps us understand the superior efficacy associated with the combination therapy compared with ICIs alone.

## Conclusions

In this meta-analysis, dual therapy was evaluated in patients who were treatment naive with liver function scale Child-Pugh A, ECOG performance status 0 to 1, and treated gastroesophageal varices. The efficacy of dual therapy in patients previously treated with VEGF inhibitors, like sorafenib or lenvatinib, is not known. Therefore, atezolizumab plus bevacizumab should be preferentially used in the first-line setting in eligible patients. The best choice after unsuccessful dual therapy is not clear. In the past few years, several options for systemic therapy have been incorporated in the clinical management of advanced HCC, raising questions about how to better sequence the drugs. Until RCTs evaluating systemic therapy after unsuccessful treatment with ICIs in combination become available, offering tyrosine kinase inhibitors is plausible. If patients are not eligible to receive bevacizumab as first-line therapy, the safety profile of ICIs as monotherapy favors immunotherapy compared with sorafenib. Several ongoing RCTs are addressing the role of combination therapies in the first-line and second-line settings, which offers promise for better outcomes in patients affected by such a severe disease.

## Notes

Supplement. **eFigure 1.** Funnel Plot for the Data From Overall Survival **eFigure 2.** Assessment of Grade 3 or 4 Treatment-Related Adverse Events
