# Supplementary material for: Efficacy and Safety Associated With Immune Checkpoint Inhibitors in Unresectable Hepatocellular Carcinoma: A Meta-analysis
Source: JAMA Netw Open. 2021 Dec 6;4(12):e2136128. doi: 10.1001/jamanetworkopen.2021.36128 (PMC8649834; doi:10.1001/jamanetworkopen.2021.36128)
Supplement: Supplement. — eFigure 1. Funnel Plot for the Data From Overall Survival eFigure 2. Assessment of Grade 3 or 4 Treatment-Related Adverse Events [file jamanetwopen-e2136128-s001.pdf]

Supplemental Online Content

Jácome AA, Castro ACG, Vasconcelos JPS, et al. Efficacy and safety associated with immune checkpoint inhibitors in unresectable hepatocellular carcinoma: a meta-analysis. *JAMA Netw Open*. 2021;4(12):e2136128.
doi:10.1001/jamanetworkopen.2021.36128

eFigure 1. Funnel Plot for the Data From Overall Survival
eFigure 2. Assessment of Grade 3 or 4 Treatment-Related Adverse Events

This supplemental material has been provided by the authors to give readers additional information about their work.

© 2021 Jácome AA et al. *JAMA Network Open.*

eFigure 1. Funnel Plot for the Data From Overall Survival

Abbreviations: SE: standard error

The *x*-axis is the hazard ratio and the *y*-axis is the standard error of the log of the hazard ratio. Each circle represents the respective hazard ratio of each study included in the meta-analysis. The vertical dashed line is the funnel axis, which represents the overall hazard ratio (0.75) of the overall survival. All the studies are scattered close to the funnel axis, which means that there is no evidence of publication bias.

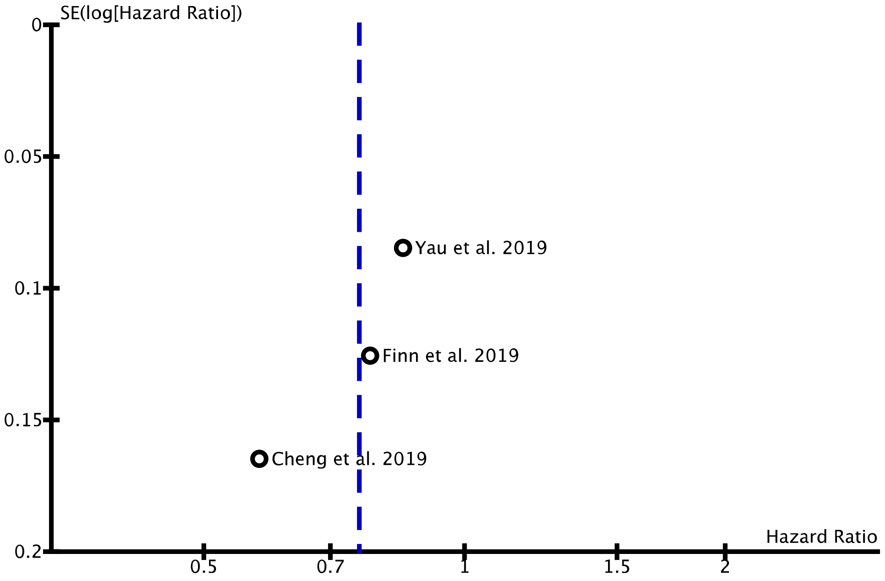

eFigure 2. Assessment of Grade 3 or 4 Treatment-Related Adverse Events

Study or Subgroup	ICI Events	ICI Total	Standard of care Events	Standard of care Total	Weight	Odds Ratio M-H, Random, 95% CI
Yau et al. 2019 (Ref. 13)	81	371	179	372	51.0%	0.30 [0.22, 0.41]
Cheng et al. 2019 (Ref. 15,16)	117	329	71	156	49.0%	0.66 [0.45, 0.97]
**Total (95% CI)**		**700**		**528**	**100.0%**	**0.44 [0.20, 0.96]**
Total events	198		250			

Heterogeneity: Tau² = 0.28; Chi² = 9.40, df = 1 (P = 0.002); I² = 89%
Test for overall effect: Z = 2.08 (P = 0.04)

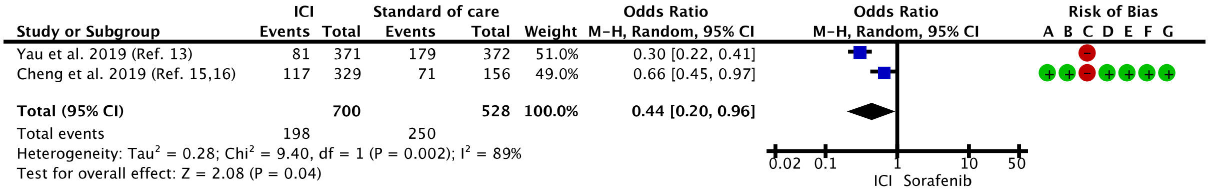

Risk of bias legend
(A) Random sequence generation (selection bias)
(B) Allocation concealment (selection bias)
(C) Blinding of participants and personnel (performance bias)
(D) Blinding of outcome assessment (detection bias)
(E) Incomplete outcome data (attrition bias)
(F) Selective reporting (reporting bias)
(G) Other bias

Abbreviations: ICI: Immune checkpoint inhibitor

© 2021 Jácome AA et al. *JAMA Network Open.*
